# Spinning-Spot Shadowless TIRF Microscopy

**DOI:** 10.1371/journal.pone.0136055

**Published:** 2015-08-26

**Authors:** Kyle L. Ellefsen, Joseph L. Dynes, Ian Parker

**Affiliations:** 1 Department of Neurobiology & Behavior, University of California Irvine, Irvine, CA, 92697, United States of America; 2 Department of Physiology & Biophysics, University of California Irvine, Irvine, CA, 92697, United States of America; University of Manchester, UNITED KINGDOM

## Abstract

Total internal reflection fluorescence (TIRF) microscopy is a powerful tool for visualizing near-membrane cellular structures and processes, including imaging of local Ca^2+^ transients with single-channel resolution. TIRF is most commonly implemented in epi-fluorescence mode, whereby laser excitation light is introduced at a spot near the periphery of the back focal plane of a high numerical aperture objective lens. However, this approach results in an irregular illumination field, owing to interference fringes and scattering and shadowing by cellular structures. We describe a simple system to circumvent these limitations, utilizing a pair of galvanometer-driven mirrors to rapidly spin the laser spot in a circle at the back focal plane of the objective lens, so that irregularities average out during each camera exposure to produce an effectively uniform field. Computer control of the mirrors enables precise scanning at 200 Hz (5ms camera exposure times) or faster, and the scan radius can be altered on a frame-by-frame basis to achieve near-simultaneous imaging in TIRF, widefield and ‘skimming plane’ imaging modes. We demonstrate the utility of the system for dynamic recording of local inositol trisphosphate-mediated Ca^2+^ signals and for imaging the redistribution of STIM and Orai proteins during store-operated Ca^2+^ entry. We further anticipate that it will be readily applicable for numerous other near-membrane studies, especially those involving fast dynamic processes.

## Introduction

The advent of total internal reflection fluorescence (TIRF) microscopy has greatly facilitated the imaging of cellular structures and processes in or very close to the plasma membrane [1]. In essence, TIRF works by exciting fluorescence restricted to a very thin sheet (of the order of 100 nm) in an aqueous medium immediately above the cover glass. Thus, TIRF microscopy provides an optical sectioning effect analogous to confocal microscopy, but by a completely different mechanism and with a much thinner section. Moreover, in contrast to the raster spot scanning of confocal microscopy, the entire section is illuminated at the same time, enabling fast camera-based imaging of dynamic processes.

TIRF works by directing excitation light through a glass substrate toward an aqueous specimen at a sufficiently shallow angle so that total internal reflection occurs due to the refractive index decrease at the glass–water interface. However, a very thin electromagnetic field (evanescent wave) is created in the liquid with the same wavelength as the incident light, which decays exponentially with distance from the interface (typically over one or a few hundred nm) [2,3]. This field excites fluorophores near the interface while avoiding excitation further into the aqueous phase. It can thus be used to selectively image fluorescence signals arising from labels and functional probes at, or in close proximity to the plasma membrane of a cell adhering to the coverslip. For example, we and others have used TIRF microscopy in conjunction with Ca^2+^ indicator dyes for fast dynamic imaging of local cytosolic Ca^2+^ transients generated by ion channels in the plasma membrane [4,5], and by channels in closely apposed regions of the endoplasmic reticulum [6].

TIRF microscopy is typically implemented by introducing a laser beam focused to a single spot at the back focal plane of a specialized, high numerical aperture (NA) objective lens [3]. The angle of the light emerging from the objective depends upon the radial position of the laser spot, and by adjusting this to the edge of the aperture the critical angle can be exceeded to achieve total internal reflection at the glass/water interface. An evanescent field is formed which decays exponentially with distance from the interface, following an exponential constant that can, in theory, be predicted from the wavelength of the light and the angle of incidence. However, when imaging in cells the presence of organelles with differing refractive indices perturbs the TIRF illumination field, resulting in marked streaks and shadows emanating from the side at which the laser light is introduced. Additional nonuniformities in excitation intensity arise from scattering and diffraction of the coherent laser light at the numerous surfaces in the optical path. Together, these processes result in highly irregular illumination across the imaging field [7,8], which obscures true image details and complicates the quantification and comparison of fluorescence signals arising at different locations. The spatial differences in excitation intensities may be partially corrected by normalizing images with respect to a previously captured flat-field image. As applied to imaging of cytosolic Ca^2+^ signals, this is typically done by taking a ratio of stimulus-evoked Ca^2+^-dependent changes in fluorescence at a pixel relative to the mean resting fluorescence at that pixel. Nevertheless, this normalization is imprecise, and cannot correct for temporal fluctuations resulting from factors including organelle movement, mechanical drift and vibrations, and ongoing, constitutively active changes in fluorescence. Moreover, uneven illumination also introduces disparities in noise levels between neighboring cellular regions, which cannot be corrected by normalization.

Several designs have been published which mitigate these problems and produce a more even illumination field by either introducing light through a continuous annular ring at the back focal plane of the TIRF objective lens [9,10], or by rapidly rotating a laser spot around this annulus [7,11,12]. We describe here a simple design of ‘shadowless’ TIRF microscope that utilizes a pair of galvanometer mirrors to spin the laser spot around a circle at the outer edge of the objective’s back focal plane. Consequently, a collimated beam with fixed polar angle and spinning azimuthal angle illuminates the specimen. The different shadowing and interference patterns at each angle thus average out during the single-frame exposure time of the camera to produce a substantially uniform illumination field [7]. Owing to the use of fast, computer-controlled galvanometer mirrors this approach is compatible with rapid (hundreds of frames per second) image acquisition. Moreover, the system is highly flexible as the scanning mode can be modulated ‘on-the-fly’ to achieve almost simultaneous (alternating frame) acquisition in regular (stationary spot) TIRF, spinning-spot TIRF and widefield fluorescence modes. We demonstrate the utility of this system for shadow-free imaging of cellular structures, and for enhanced recording of dynamic processes including local Ca^2+^ signals in cultured cells and two-color imaging of the relocalization of STIM and Orai proteins during activation of store-operated Ca^2+^ entry.

## Materials and Methods

### Microscope design

**Fig 1:**
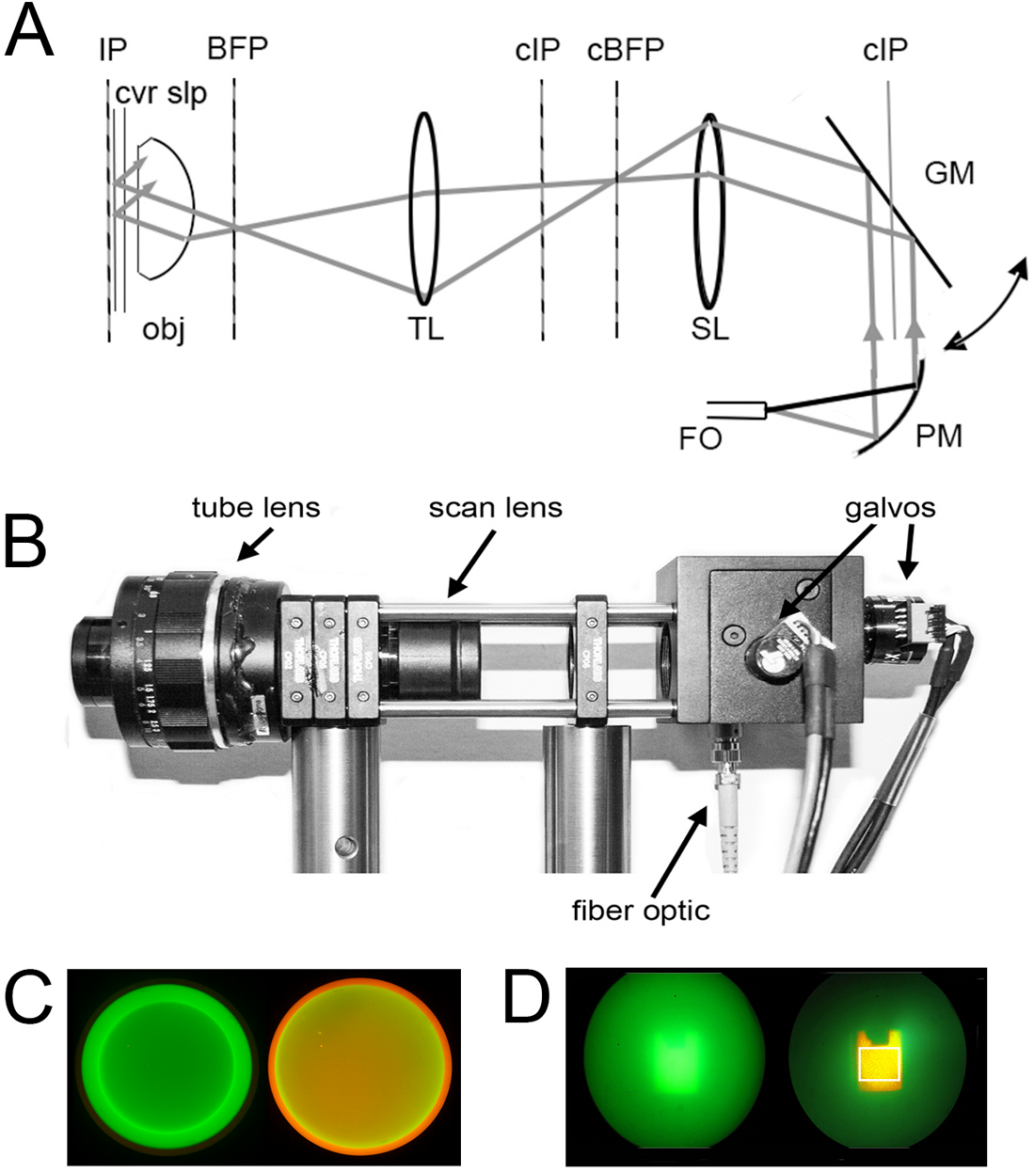
Optical scheme of the spinning spot TIRF illuminator. (A) Optical schematic. Laser light from a single-mode fiber optic (FO) is collimated by a parabolic mirror beam expander (PM) and directed onto a pair of orthogonal galvanometer-driven mirrors (GM: only a single mirror is shown for clarity). Reflected light is relayed through a scan lens (SL: formed by a 10x eyepiece) and a tube lens (TL: formed by a 50 mm *f* 1.8 camera lens) which focus the laser light to a diffraction-limited spot at the back focal plane (BFP) of the microscope objective (obj). A dichroic mirror placed between the tube lens and objective (not shown) reflects laser light to the objective, while allowing emitted fluorescence to pass through to the imaging camera. IP = image plane; cIP = conjugate image planes; cBFP = conjugate back focal plane. (B) Photograph of the spinning spot TIRF illuminator, constructed from Thor Labs optical cage components. (C, D) Photographs taken through the microscope ocular, illustrating the alignment procedure using a rhodamine film/fluorescein solution test specimen. (C) Images taken with a Bertrand lens present so as to view the back focal plane of the objective lens. The left panel shows the narrow green circle traced by the spinning lase spot when the radius is set below the critical angle for total internal reflection. The right panel was captured after increasing the scan radius to achieve TIRF excitation. The appearance of the orange ring arises from selective fluorescence excitation of the film of rhodamine within the evanescent field. (D) Corresponding appearance of the fluorescein solution/rhodamine film test specimen viewed conventionally (i.e. without the Bertrand lens) with the radius of the scan circle below (left) and above (right) that required for TIRF excitation. The white box in the right panel outlines the approximate imaging field of the EMCCD camera.

The optical scheme (Fig 1A) is designed to focus a laser beam to a spot at the back focal plane (BFP) of the microscope objective, at an off-axis position the azimuthal angle of which can be rapidly changed. The tube lens (TL) forms conjugate planes in free space (cIP, cBFP respectively) to the image plane of the sample (IP) and the back focal plane. Beams from 488 nm and 532 nm solid-state lasers are combined by a dichroic beamsplitter (Semrock 503nm LaserMUX) and coupled into the TIRF illuminator through a single-mode fiber optic (FO), from which the divergent beam is collimated into an 8mm wide beam by an off-axis parabolic mirror (PM). This beam over-fills a pair of mirrors on orthogonally-mounted galvanometers, which are driven by 90° phase-shifted sine waves so that the emerging light scans in a circular pattern. Relay lenses SL and TL direct the laser light via a reflecting dichroic mirror (omitted for clarity in Fig 1A) so that it is focused to a spot at the back focal plane of the microscope objective (obj: Olympus 60x NA 1.45 TIRF objective). The spot is rotated by the action of the scan mirrors, and by adjustment of the drive waveforms is made to describe a circle centered on the optical axis and with a radius near the outer edge of the objective back aperture such that the laser light undergoes total internal reflection at the interface between the coverglass and aqueous specimen at all positions around the circle. When correctly adjusted, a near-stationary image of the scan mirrors is cast at the specimen plane, forming the TIRF-illuminated imaging field. Using 4 mm galvanometer mirrors and the optics shown in Fig 1A the usable imaging field is a square of about 50 μm side. A complete components list can be found in S1 Supporting Information.

### Microscope construction

Fig 1B shows a photograph of the TIRF illuminator, which is used in conjunction with an Olympus IX70 microscope fitted with a 60x NA1.45 TIRF objective lens. The TIRF illuminator is a free standing unit, which in our instrument interfaces with the microscope via a laser port formed by a side-pointing filter cube containing a 500 nm dichroic mirror and 510nm long-pass emission filter. Alternatively, it would be possible to interface the illuminator via the rear port of the microscope if this were not otherwise occupied. Our microscope is further equipped with a second, upper port, used for UV flash photolysis of caged compounds. The illuminator was constructed using standard Thor Labs fiber optic, galvanometer mirror, cage and mounting components. The scan lens (SL) is an Olympus 10x eyepiece with focusing mount, and the tube lens (TL) is a 50 mm f1.8 SLR camera lens.

### Software

The galvanometers are driven by analog signals generated by a National Instruments DAC PCI-6733 card in a PC running Windows 7. A custom program developed in the open source programming language Python enables control via a graphical user interface (S1 Fig). A master control sets the amplitude of signals to both galvanometers (i.e. the radius of the circle), and the offset (center of the circle) can be adjusted in *x* and *y* directions. Moreover the ratio between amplitudes of the sine and cosine waves can be further adjusted to alter the ellipticity of the scan (ratio of width to height), and their respective phase (width along diagonal axes) so as to compensate for any astigmatism in the optics and differences in sensitivity and phase lag between the galvanometer systems. Once a setting is adjusted, a lookup table for a sine and cosine wave with user defined frequencies and 1MHz sample rates is generated and stored on the DAC. In addition to the analog outputs controlling the galvanometer scanners, the software also provides TTL (on/off) and analog (variable power) outputs to control two different lasers (488 nm and 532 nm DPSS lasers in our system) to enable two-color imaging with fluorophores of differing excitation spectra. With the exception of the STIM/Orai relocalization experiment, all data presented here were obtained using 488 nm excitation. All of these settings can be saved as presets, and can be recalled by clicking one of three individual buttons that can be configured to achieve different fluorescence excitation modes: for example, shadowless TIRF (with the circular scan falling in the annular region of the objective aperture producing total internal reflection); regular TIRF (with the laser spot at any stationary position within this annulus); ‘skimming-plane’ illumination (with the circular scan at a radius just smaller than that required for TIRF, so that the emerging laser beam skims just above the cover glass); or pseudo-widefield illumination (with a circular scan appreciably smaller than the radius needed for total internal reflection). Moreover, the settings from up to three presets can be interleaved, frame by frame, to accomplish near-simultaneous imaging by different modalities (e.g. TIRF and pseudo-widefield), and/or excitation of different fluorophores by different laser lines.

### Image acquisition and processing

**Fig 2:**
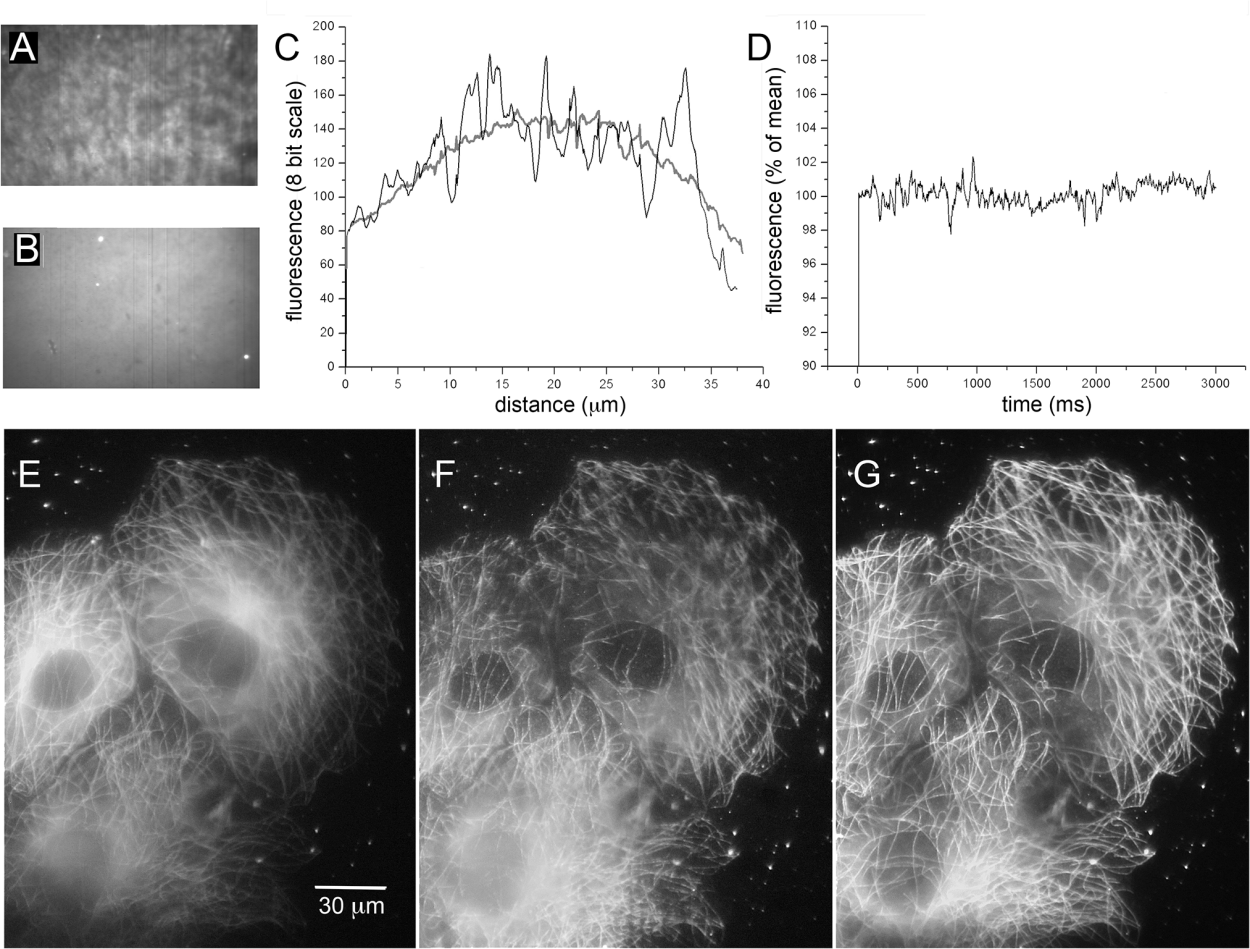
Improved field uniformity with shadowless TIRF excitation. (A, B) Images (500x300 pixel, 40 x 24 μm) of rhodamine film on a coverglass. The image in A was captured using stationary-spot (conventional) TIRF excitation at 488 nm; that in B shows the same specimen but imaged by spinning-spot TIRF excitation. Dirt specks and fluorescent beads are readily discerned in B, but are almost completely masked by irregularities in the excitation field in A. The grey-scale levels in each panel were adjusted to equalize black levels and the brightest regions (excluding the fluorescent beads in B). (C) Measurements of fluorescence intensity along horizontal lines drawn across the center of the images in A (irregular black trace) and B (grey trace). (D) Stability of fluorescence excitation over time. The plot shows mean fluorescence intensity within a 100 x 100 pixel region in the center of the field, measured from 600 image frames acquired at 5 ms intervals in spinning-spot TIRF mode. (E-G) Representative images showing COS-7 cells expressing tubulin tagged by EGFP, captured using wide-field excitation (E), by stationary-spot TIRF (F) and spinning-spot TIRF (G). Images were captured with 488 nm excitation during 2s exposures using an 18 Mpixel Canon EOS M camera and eyepiece adapter.

Electron-multiplied CCCD (emCCD) cameras (Cascade, Roper Scientific) were used for acquisition of dynamic fluorescence signals; a Cascade 128 for fast (5 ms exposure time) imaging of Ca^2+^ signals, and a Cascade 650 to provide greater pixel resolution for slower (500 ms exposure time) imaging of STIM/Orai dynamics. By utilizing a TTL pulse generated by the scanner software to initiate acquisition by the camera, each frame represents an average across all 360° azimuthal incidence angles, producing the effect of a spatially and temporally uniform field of illumination. High resolution and color images (Figs 1C, 1D and 2E–2G) were obtained using a 35mm digital camera (Canon EOS M) with long (2s) exposures to summate over hundreds of scan cycles.

Images from the Cascade emCCD cameras were acquired using MetaMorph (Universal Imaging/ Molecular Dynamics), but other software, including open-source packages such as Micro˗Manager (www.micro-manager.org) would be equally applicable. Processing and analysis was facilitated by a custom software package written in Python (S2 Fig). This enables black level subtraction, de-interleaving into separate image stacks (e.g. stacks representing near-simultaneous shadowless TIRF and WF images) and processing of each stack to yield pseudo-ratio (ΔF/F_0_) image stacks where the value of each pixel is normalized relative to the average value calculated from a specified range of resting frames acquired before stimulation. Duplicate regions of interest can then be defined on multiple image stacks, which are linked so that movement of one region is mirrored by its paired region on the other stack. A window displays superimposed traces over time of average pixel intensities within the paired regions, and these traces can be saved as ASCII files for subsequent analysis and plotting.

The open-source software routines for controlling the scan mirrors are available at https://github.com/kyleellefsen/shadowlessTIRF. Open-source software used for image processing is available at https://github.com/kyleellefsen/Flika.

### Initial set-up

When first installing the TIRF illuminator, it must be set so the optical axis is parallel to and collinear with the optical axis of the microscope; that is to say, with the scan mirrors centered, the laser spot should appear centered on the objective lens. The distances from the objective to the tube and scan lenses and to the scan mirrors are adjusted so that the laser beam is focused to a spot at the back focal plane of the objective. Focusing so that the beam projected from the objective lens forms the smallest spot directly on the ceiling above can easily ensure this. Further, the position of the scan mirrors is adjusted so that they are re-imaged onto the specimen plane, such that rotation of the mirrors causes minimal movement at the specimen plane.

To assist in alignment, we use a test specimen made from an aqueous solution of fluorescein (2 μM) and rhodamine (1 μM) in a culture dish with a cover glass as its base (Mattek). The fluorescein remains in free solution, while the rhodamine quickly adheres as a thin film to the coverglass. Thus, when illuminated by wide-field epifluorescence a diffuse green glow is seen through the eyepiece as the laser light excites fluorescein throughout the bulk (a few mm deep) solution. In contrast, when adjusted for TIRF illumination, the film of rhodamine is selectively excited, producing a distinct orange fluorescence outlining the image of the scan mirror. When correctly aligned, displacement of a stationary (non-scanning) laser spot toward the edges of the objective aperture in both *x* and *y* axes produces an abrupt transition into TIRF illumination, revealing a distinct orange rectangle which should remain in about the same position at each extreme setting (top-bottom and left-right) of the laser spot.

### Routine alignment for shadowless TIRF

Correct alignment of the spinning-spot is most easily accomplished by use of a Bertrand lens (‘centering telescope’) that allows an image of the back focal plane of the objective to be viewed through the microscope. The microscope should be focused on the fluorescein/rhodamine test dish described above. With the scan radius set well below that giving TIRF illumination, a green circle will be apparent when viewed through the Bertrand lens (Fig 1C, left). Adjustment of the x, y position and ellipticity and phase sliders can be used to center the scan within the outline of the objective aperture, and to ensure that it is almost perfectly circular. Increasing the scan radius will then make the circle enlarge and eventually reach the critical angle for TIRF excitation. At that point the central circle will dim and become more orange and a bright orange/yellow outer rim will become evident; both features originating from selective excitation of the rhodamine film within the evanescent field (Fig 1C, right). Fig 1D shows corresponding images of the specimen viewed directly through the eyepiece, with the Bertrand lens removed. Excitation with the scan radius below that required for TIRF excitation shows a blurred green central square, arising predominantly from fluorescence of fluorescein in the bulk solution (Fig 1D, left). In contrast, when the scan radius is increased to exceed the critical angle for total internal reflection a sharply defined orange square is seen, resulting from selective excitation of the rhodamine film on the coverglass by the evanescent wave (Fig 1D, right). Fine adjustments can then be made to optimize the laser scan. Only a diffuse and dim surrounding green glow should be apparent from the fluorescein solution, and the appearance of a green ‘comet tail’ indicates misalignment of the scan such the radial position of the laser spot falls below that required for TIRF at a circumferential position opposite the comet tail. Our experience is that, once adjusted, the alignment of the system remains stable for many days.

Note that the images in Fig 1C and 1D, show the entire field of view through the eyepieces. The central orange square in Fig 1D right defines the smaller TIRF illumination field. All other images shown here were obtained at higher magnification, zooming in on this field.

### Cell imaging

SH-SY5Y, COS-7 and HEK-293 cells were obtained from ATCC. Procedures for imaging Ca^2+^ puffs were as described [13]. In brief, SH-SY5Y cells were loaded with the fluorescent Ca^2+^ indicator Cal-520, caged iIP_3_ and the slow Ca^2+^ buffer EGTA by incubation with membrane permeant esters of these compounds. Cells were imaged in a Ca^2+^-free medium at room temperature, and Ca^2+^ puffs were evoked by photoreleasing iIP_3_ by flashes of UV light focused uniformly throughout the imaging field. Images of tubulin in Fig 2 were obtained from COS-7 cells that were transfected 3 days before imaging with Lipofectamine 2000 (Invitrogen) using EGFP-tubulin plasmid (Addgene). Imaging of STIM/Orai translocation was done using human embryonic kidney (HEK) 293A cells (Invitrogen) incubated at 37°C, 5% CO_2_ and maintained in Dulbecco’s modified Eagle’s medium (DMEM; Lonza) supplemented with 10% fetal calf serum (Omega scientific) and 2 mM L-glutamine (Sigma-Aldrich). HEK cells were transfected with Lipofectamine 2000 (Invitrogen) as directed by the manufacturer using 2.6 μg mCherry-STIM1 and 1.3 μg EGFP-Orai1 per well of a 6-well petri dish. Cells were split the following day into 35 mm MatTek dishes with integrated coverslip bottoms coated with poly L-lysine (>300K MW, 0.1 mg/ml in water; Sigma-Aldrich) and were imaged at room temperature in Ringer’s solution on the second day after transfection. Calcium store depletion was induced by addition of 2 μM thapsigargin (Calbiochem). The plasmid mCherry-STIM1 was constructed using an Infusion kit (Clontech) based cloning approach. Starting with the plasmid YFP-STIM1 [14], the yellow fluorescent protein was replaced with the red fluorescent protein mCherry. This fusion mCherry-STIM1 gene was ligated into the expression plasmid pmaxGFP (Lonza), replacing the GFP gene.

## Results

### Shadowless illumination by spinning-spot TIRF

Fig 2 illustrates various specimens, demonstrating the improved spatial uniformity of illumination achieved by spinning-spot TIRF excitation as compared to conventional, stationary-spot TIRF. Fig 2A–2C show images of a test specimen made by depositing a film of rhodamine and sparsely distributed 200 nm red fluorescent microspheres from an aqueous solution onto a coverglass. Fig 2A was imaged using conventional TIRF excitation with the laser spot stationary at the outer edge of the objective aperture. The field is highly irregular, patterned as a result of interference of the coherent laser light at various interfaces and imperfections in the optical path. Pixel intensities varied by as much as ± 30% between adjacent regions. For comparison, Fig 2B shows the same specimen, now imaged by spinning-spot TIRF. The field is much more uniform, and it is possible to resolve features and fluorescent beads in the specimen that were almost completely obscured in the conventional TIRF image. The improvement in field uniformity is quantified in Fig 2C, showing linescan measurements of fluorescence intensity across the center of the images in Fig 2A and 2B. The plot for the spinning-spot TIRF image (thick grey curve, Fig 2C) shows a relatively smooth distribution, defined by the Gaussian profile of the laser beam, whereas the plot for the stationary-spot TIRF image is highly irregular (black trace, Fig 2C).

For dynamic measurements, such as Ca^2+^ signals, it is important that fluctuations in fluorescence excitation are minimal so that they contribute little noise above the irreducible limit imposed by photon shot noise. Fig 2D shows fluorescence emission from a rhodamine film imaged over 3s by spinning-spot TIRF. Measurements were taken over a wide (100 x 100 pixel) region to average out shot noise, and show that the laser excitation incident on the specimen remained constant within about ± 1% of the mean.

Fig 2E–2G further illustrate the advantages of spinning-spot TIRF for imaging cellular structures. The three panels show the same group of COS-7 cells, transfected to overexpress EGFP-tagged tubulin. Cells were imaged in wide-field mode (Fig 2E), stationary-spot TIRF (Fig 2F), and spinning-spot TIRF (Fig 2G). Both TIRF images display much less out-of-plane background fluorescence than the wide-field image, isolating those microtubules lying close to the coverglass. Moreover, the spinning-spot TIRF image (Fig 2G) reveals finer details with more even illumination across the field, whereas the conventional, stationary-spot TIRF image (Fig 2F) is marred by patterning of the fluorescence excitation and by highlighted and shadowed areas where the unidirectional laser beam has been perturbed by cellular structures.

### Imaging local Ca^2+^ signals by spinning-spot TIRF

Ca^2+^ puffs are localized subcellular Ca^2+^ signals evoked by Ca^2+^ liberation from the ER through inositol trisphosphate (IP_3_) receptors. They have durations of several tens to a few hundred ms, so that imaging frame rates of hundreds of Hz are required to resolve kinetic details during individual puffs. We had previously described that TIRF imaging provides enhanced resolution of puffs arising at sites near the plasma membrane [13], but the highly irregular excitation field of conventional (stationary-spot) TIRF illumination complicates the identification of puff sites and quantification of fluorescence amplitudes. Although these issues can be mitigated by forming ratio images of fluorescence relative to the mean fluorescence at each pixel under quiescent conditions, problems remain in that the signal-to-noise ratio is degraded for events at sites that are shadowed, and it may be difficult to obtain ‘resting’ baseline images in cells that show constitutive activity. We thus explored the use of spinning-spot TIRF to image puffs with improved spatial uniformity of fluorescence excitation.

**Fig 3:**
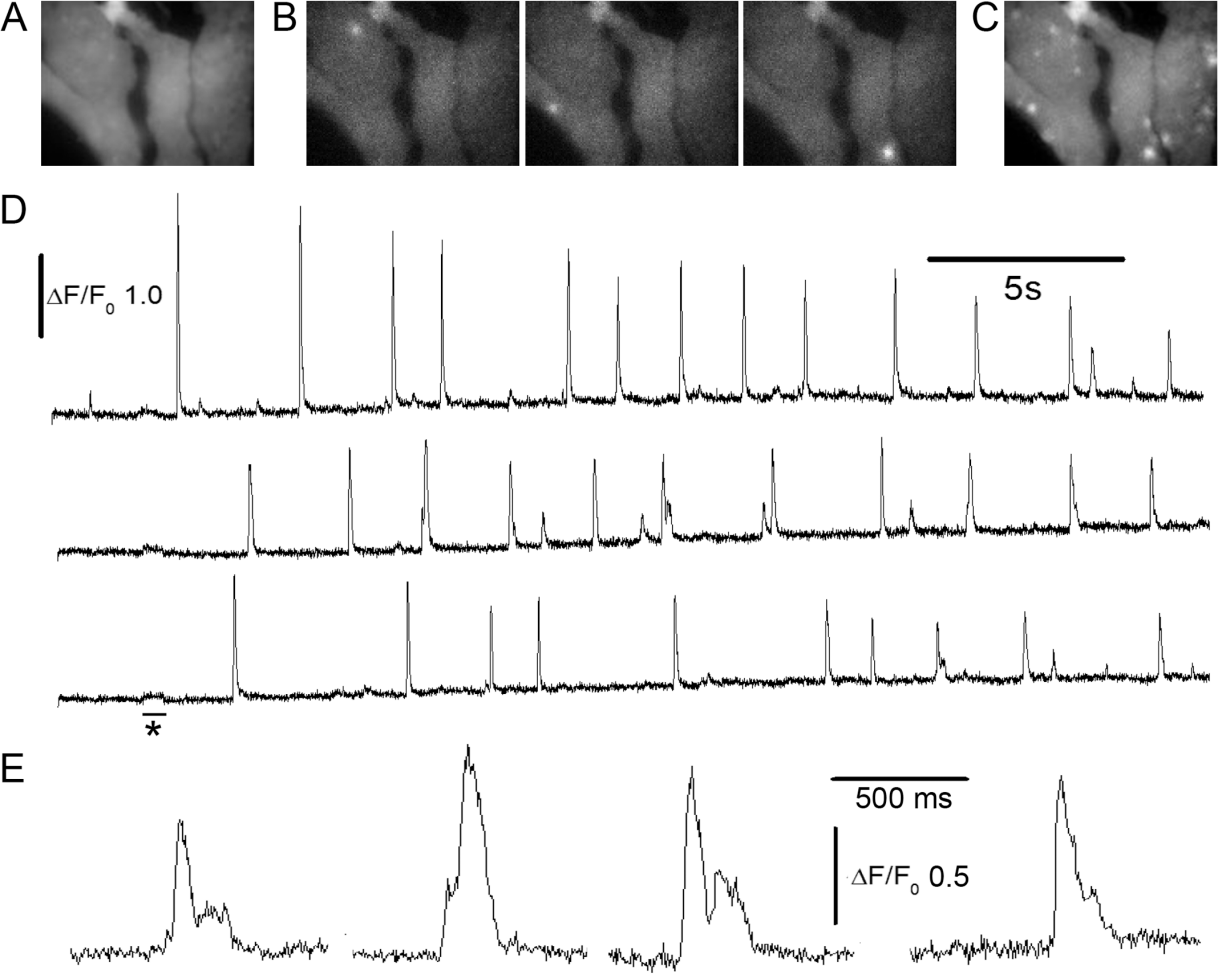
Imaging local Ca^2+^ puffs by shadowless TIRF. (A) Averaged image (mean of 100 frames; 51 x 38 μm) of resting fluorescence in SH-SY5Y cells loaded with Cal520. (B) Representative examples of individual image frames captured at different times after photorelease of IP_3_, illustrating puffs arising at three different locations in the cells. (C) Maximum intensity profile across 5000 image frames acquired following photorelease of IP_3_ showing sites of puff activity in the cells. (D) Traces show fluorescence ratio measurements (ΔF/F_0_) derived from small (~1x1 μm) regions of interest centered on the puff sites shown in (B). (E) Selected examples of events shown on an expanded timescale to illustrate temporal resolution of stepwise transitions during puffs.

We imaged puffs in SH-SY5Y neuroblastoma cells loaded with the fluorescent Ca^2+^ indicator Cal-520 together with caged iIP_3_ and the slow Ca^2+^ buffer EGTA [13]. Cells imaged by spinning-spot TIRF illumination (488 nm) before stimulation showed a relatively uniform resting fluorescence. (Fig 3A) Photorelease of iIP_3_ by a UV light flash delivered within the imaging field then evoked Ca^2+^ release from the ER through IP_3_ receptors, which were apparent as transient localized events (puffs) arising discrete sites because the EGTA suppressed Ca^2+^-induced Ca^2+^ release between adjacent clusters (Fig 3B). A maximum intensity projection of fluorescence across 5000 frames (12.5s) showing persistent puff activity clearly revealed several puff sites, superimposed on the background fluorescence (Fig 3C). Representative traces of fluorescence ratio (ΔF/F_0_) from three puff sites in Fig 3D and 3E, illustrate the low noise and good temporal resolution of these recordings.

### Fast dynamic imaging in interleaved modes

A particular advantage of utilizing computer-controlled galvanometer mirrors to scan the laser spot is that effectively simultaneous image sequences can be captured in different excitation modes by alternating exposures with different scan settings. One example of this is illustrated in S1 Movie, which shows fluorescent beads both in suspension and adherent to the cover glass. Image frames were captured at 5 ms intervals, alternating frames between spinning spot TIRF mode and a pseudo widefield mode obtained by setting the scan radius well below that required to achieve total internal reflection. The widefield images show beads undergoing Brownian motion in three dimensions in the aqueous medium, whereas the TIRF images show only those beads adhering to the coverglass or approaching close to it.

**Fig 4:**
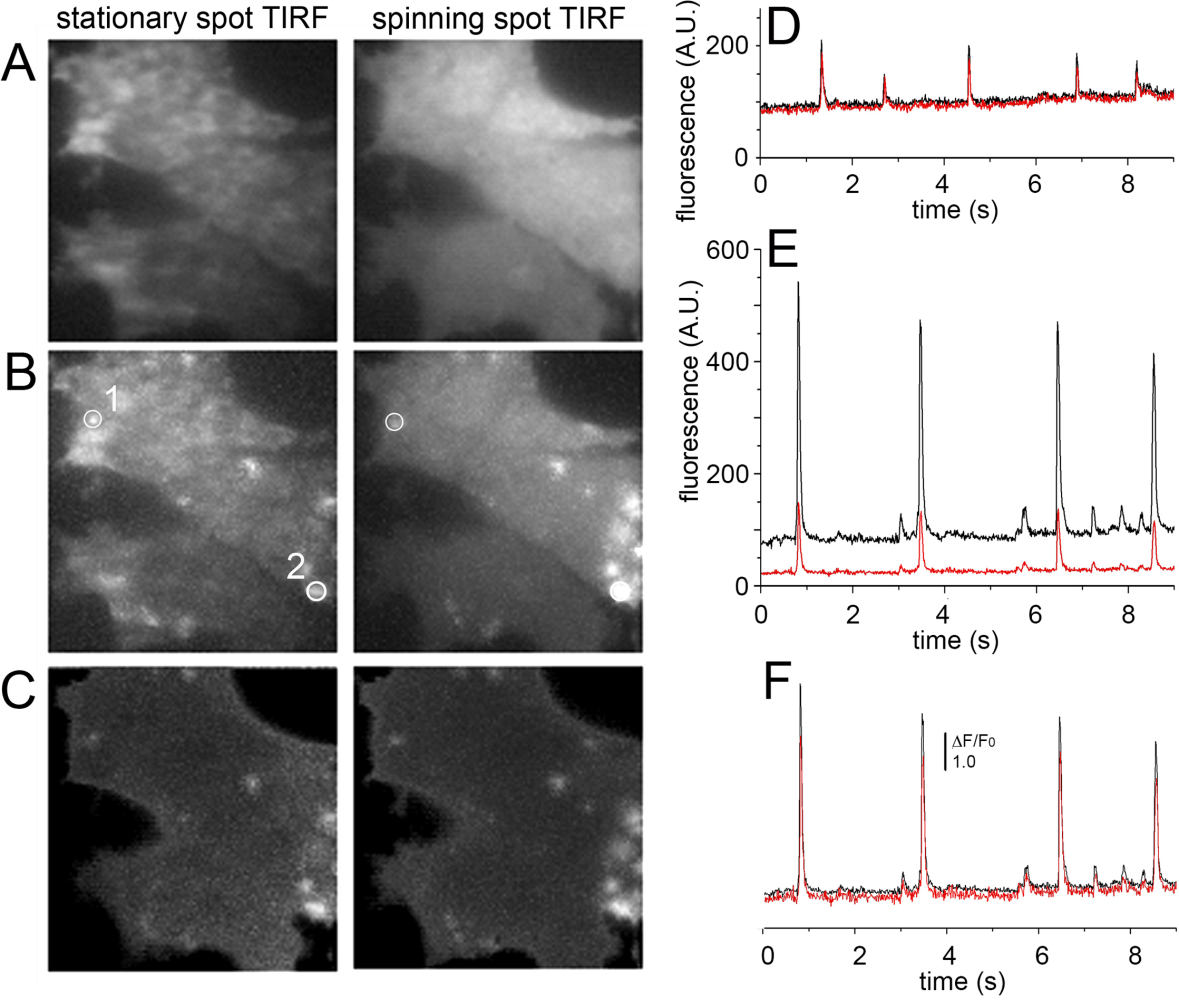
Interleaved stationary- and spinning-spot TIRF imaging of Ca^2+^ puffs. Alternate 5ms exposures were made in each mode, and image panels and traces were derived after de-interlacing the image sequence. Image panels acquired in stationary spot (left) and spinning spot (right) modes show (A), resting fluorescence (F_0_) averaged from 100 frames before photorelease of IP_3_; (B), maximum intensity projections of fluorescence (F) during 900 frames after photoreleasing IP_3_ to evoke puff activity; (C), maximum intensity projections of fluorescence ratio changes (ΔF/F_0_) during the same 900 frames. Image size is 40 x 40 μm. (D, E) Traces showing fluorescence measured, respectively, from the regions of interest marked as 1,2 in the panels in (B). In each case, the black trace was obtained in spinning spot mode, and the red trace in stationary spot mode. (F) Traces as in (E), after normalizing as fluorescence ratio (ΔF/F_0_) measurements.

Fig 4 further illustrates a comparison between stationary- and spinning-spot TIRF modes for fast dynamic imaging of Ca^2+^ puffs. Images were acquired as in Fig 3, excepting that alternating frames (5 ms exposure time) were acquired with the laser spot stationary at the periphery of the circular scan (Fig 4A–4C, *left*) and in spinning spot TIRF mode (Fig 4A–4C, *right*). The resting, background fluorescence of cells imaged by spinning spot TIRF was spatially quite uniform, but was highly irregular with stationary spot excitation (Fig 4A). Maximum intensity projections across 900 frames acquired after photoreleasing IP_3_ to evoke Ca^2+^ puffs showed clearly delineated puff sites in the spinning spot recording, whereas many of these sites were obscured and hard to identify in face of the irregular background in the stationary spot recording of ‘raw’ fluorescence (Fig 4B). Although the spatial irregularities in illumination by stationary spot TIRF could be substantially normalized by forming ratio images relative to an average of resting fluorescence before stimulation (ΔF/F_0_), maximum value projections of ΔF/F_0_ ratio images remained less clearly defined and failed to reveal some events captured in ΔF/F_0_ projections from spinning spot illumination (Fig 4C). Fig 4D and 4E show representative recordings of ‘raw’ fluorescence from the two regions of interest marked in Fig 4B; in each case the red trace was recorded with stationary spot illumination and the black trace with spinning spot illumination. The region in Fig 4D was relatively bright in the stationary spot image, and the two fluorescence traces closely overlay. In contrast, the region in Fig 4E was shadowed, so that the fluorescence amplitude of puffs was considerably lower in the stationary- as compared to spinning-spot records. When expressed as ΔF/F_0_ ratios the traces became more similar, but the stationary-spot record showed an appreciably higher noise level.

### Alternating TIRF and wide-field illumination to determine depth of puff sites

We had previously described the benefit of TIRF imaging for high fidelity recording of Ca^2+^ signals arising at puff sites close to the plasma membrane of cultured mammalian cells [6,13]. However, a proportion of sites in SHSY-5Y cells lie deeper into the cell, so that fluorescence signals were diminished or lost when the imaging mode was switched from widefield fluorescence to TIRF during a recording [13]. The presence of these deeper sites raises a problem for images acquired solely in TIRF mode, in that the fluorescence signal arising from a given Ca^2+^ flux may show different amplitudes and kinetics depending on the axial location of the source. The spinning-spot illuminator now gave us the opportunity to systematically evaluate this factor, by near-simultaneous imaging of puffs in TIRF and wide-field (WF) modes.

**Fig 5:**
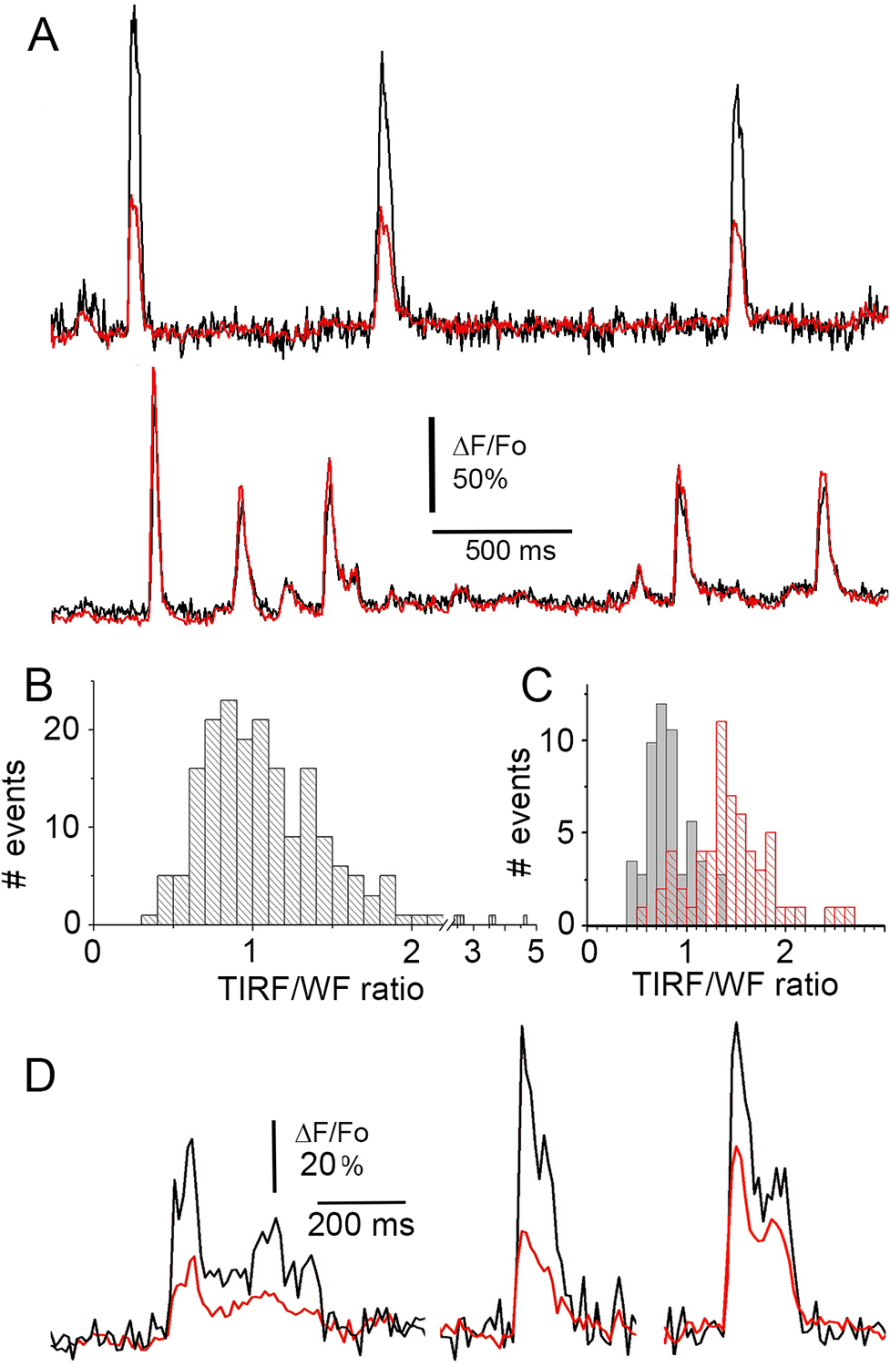
Interleaved imaging of Ca^2+^ puffs in TIRF and in wide-field modes. (A) Fluorescence recordings from two puff sites, where Ca^2+^ release originated close to the plasma membrane (top) and deeper into the cell (bottom). Each record shows fluorescence monitored in TIRF mode (black) and WF mode (red). Alternate 5ms exposures were made in each mode, and fluorescence measurements were made from 3x3 pixel (~1 x 1 μm) regions of interest centered identically over the puff site on the two de-interlaced image sequences. (B) Distribution of puff amplitude ratios as measured in TIRF and WF modes. A ratio >1 indicates that a puff gave a larger signal in TIRF than in WF mode; and vice versa for ratios <1. Data are from 189 events at sites that gave ≥4 puffs, in 14 SH-SY5Y cells. (C) Puff amplitude ratios (TIRF/WF) segregated by sites which showed mean ratios >1.1 (red) and <0.9 (black). (D) Examples of ‘superficial’ puffs imaged in interleaved TIRF (black) and WF (red) modes, shown on an expanded timescale to illustrate the improved kinetic resolution of TIRF imaging.

Fig 5A shows representative fluorescence ratio traces from two puff sites, obtained by interleaving 5ms exposures in TIRF (black traces) and WF (red traces) modes. The upper record was obtained from a site where puffs showed consistently greater amplitudes (ΔF/F_o_) in TIRF mode, whereas the lower trace illustrates a site where greater amplitudes were obtained in WF mode. We interpret the ratio of puff amplitudes obtained for each event in TIRF/WF modes as reflecting the axial location where that event arose. For example, TIRF imaging would show a small signal from a deep site because fluorescence excited in the evanescent field would be limited to the small fraction of released Ca^2+^ that diffused near the plasma membrane. Fig 5B plots the ratios of puff amplitudes simultaneously monitored by TIRF *vs*. WF imaging from 189 events. This shows a broad distribution, with roughly equal numbers of puffs showing amplitudes greater in TIRF than WF (ratio >1) and *vice versa* (ratios <1). The variability in ratios arises primarily from differences between puff sites, rather than variability among successive puffs recorded at a given site (Fig 5A). This is further illustrated in Fig 5C, where we separated ratio measurements from individual puffs into two groups, according to whether the mean ratio of all puffs (n >4) at a site was <0.9 (grey, solid histogram bars) or >1.1 (red, hatched bars). The distributions of puff ratios within each group were considerably narrower than for the all-event distribution (Fig 5B), and segregated clearly from one another.

### Triple-interlaced imaging of STIM-Orai interaction

Activation of store-operated Ca^2+^ entry involves an interaction between Orai proteins in the plasma membrane, which form a Ca^2+^-permeable pore, and STIM proteins in the ER membrane, which sense depletion of luminal Ca^2+^ levels [15]. When ER Ca^2+^ stores are replete, STIM is primarily localized deep in the cell and undergoes dynamic motions along microtubules, whereas upon store depletion it accumulates together with Orai in discrete punctae in close apposition to the cell membrane. Here, we illustrate the applicability of interleaving both imaging modes and laser wavelengths by the spinning spot TIRF system for imaging the three-dimensional relocalization of these proteins following store depletion.

**Fig 6:**
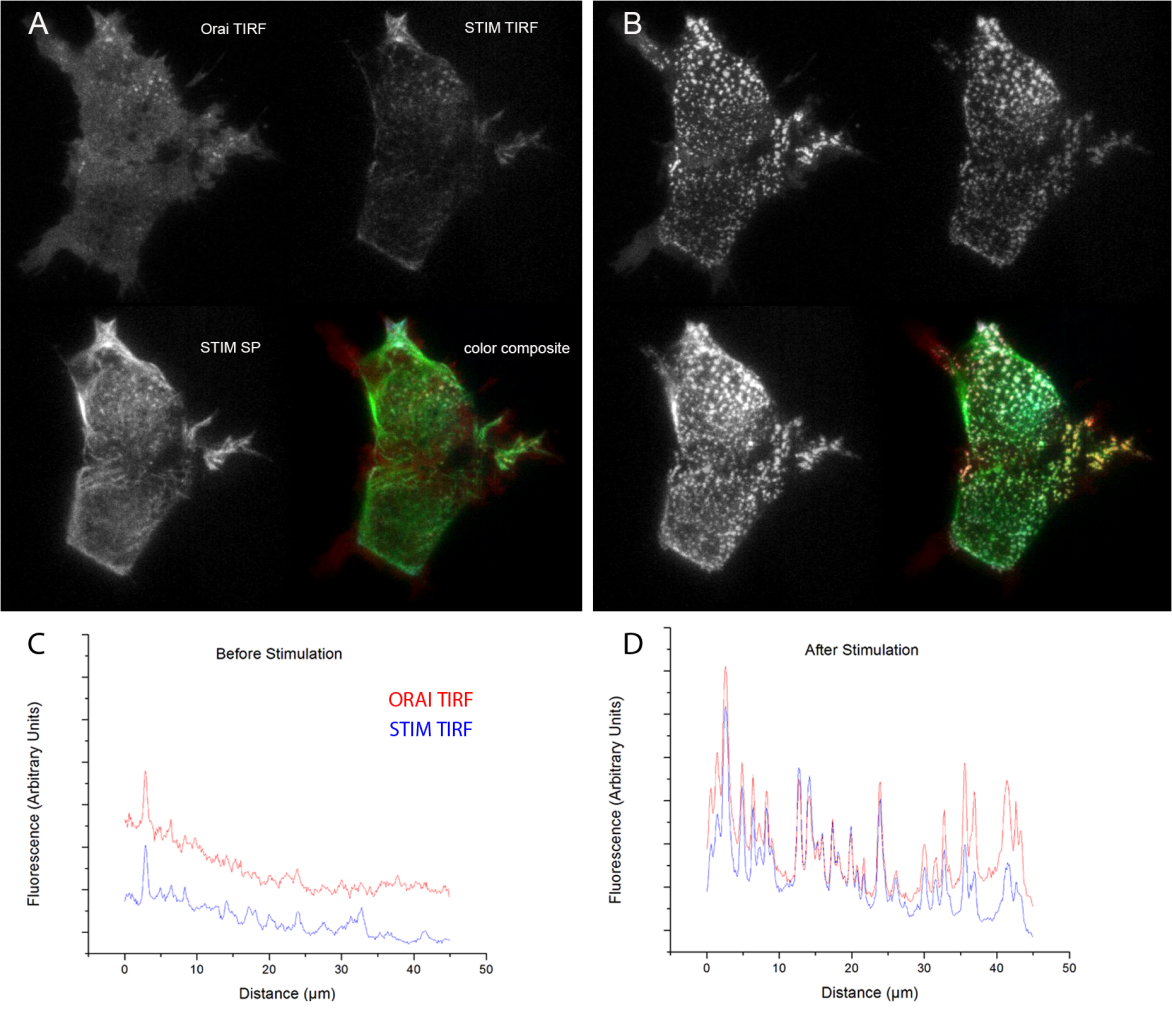
Multi-mode imaging of STIM/Orai relocalization. Images in each panel show EGFP-tagged Orai visualized by shadowless TIRF excitation at 488 nm (top left); mCherry-tagged STIM visualized by TIRF excitation at 532 nm (top right); mCherry-tagged STIM visualized by skimming- plane (SP) excitation at 532 nm (bottom left); and a pseudocolored overlay (Orai in green, TIRF-STIM in red, SP-STIM in blue) of these three images. Images were acquired before (A) and about 3 min after (B) addition of thapsigargin to deplete ER Ca^2+^ stores and induce relocalization and clustering of Orai and STIM. Each image (81 x 61 μm) is an average of two successive exposures; brightness and contrast settings were individually adjusted for each mode, but are consistent between control and thapsigargin conditions. Traces in (C) and (D) show line-scans of EGFP-Orai (red traces) and mCherry-STIM (blue traces) fluorescence in TIRF mode obtained before from 20-frame averages captured before (C) and after (D) thapsigargin treatment.

HEK 293 cells were transfected to express Orai1 tagged with EGFP together with STIM tagged with mCherry. Interleaved image frames (500 ms exposure) were acquired in TIRF mode with 488 nm excitation (to visualize Orai in the plasma membrane), in TIRF mode with 532 nm excitation (to visualize STIM in close apposition to the plasma membrane), and in skimming-plane mode with 532 nm excitation (to visualize STIM deeper into the cell). Representative images, taken from S2 Movie, are shown before (Fig 6A) and after (Fig 6B) addition of thapsigargin (1 μM) to block SERCA activity and consequently deplete ER Ca^2+^ stores. In control conditions, Orai was uniformly distributed throughout the TIRF footprint, with just a few punctae visible in the upper of the two cells (Fig 6A, top left panel; red trace, Fig 6C), but redistributed into numerous punctae after adding thapsigargin (Fig 5B, top left panel; red trace Fig 6D). At rest, STIM was evident in the skimming-plane image, moving dynamically along microtubules (Fig 6A, bottom left), whereas in the TIRF image STIM primarily restricted to the few pre-existing punctae (Fig 6A, top right; blue trace Fig 6C)). After adding thapsigargin, STIM aggregated into punctae substantially colocalized with Orai (Fig 6B, color composite; blue trace, Fig 6D). The TIRF and skimming-plane images of STIM then appeared closely similar (Fig 6B, upper right and lower left panels; Fig 6D).

## Discussion

TIRF microscopy has become the technique of choice for cellular imaging of near-membrane structures and dynamic processes [1,16]. In comparison to confocal microscopy, TIRF imaging provides substantially better axial (z) resolution and a faster time resolution than even spinning-disc confocal microscopy, limited only by camera frame rate and signal-to-noise considerations. Most TIRF microscopes, including commercial systems from the major manufacturers, employ ‘through-the-objective’ excitation, whereby laser light is introduced at a single spot near the edge of the back focal plane of a high numerical aperture TIRF objective. A drawback, however, is that the evanescent field created by light impinging at a single azimuthal angle introduces spatial non-uniformity across the imaging field owing to interference of the coherent laser light, together with shadowing and streaking caused by refractive index variations in the sample.

Filling an annular ring around the objective lens’ back focal plane to achieve substantially uniform illumination can mitigate this deleterious effect. Various approaches have been described to achieve ‘shadowless’ TIRF in this way, including an annular mask to block laser light from all but a circumferential ring [9]; by using axicon mirrors to concentrate the laser light into an annular ring [10], or by spinning an incident laser beam spot around the back focal plane using a rotating wedge [7] so that the different interference patterns at every particular azimuthal incidence angle average out over a single camera exposure. All such mechano-optical approaches are relatively inflexible in that the alignment cannot readily be changed; moreover an annular mask sacrifices most of the laser power, and the speed of a rotating wedge is limiting (a frame rate of 500 fps corresponds to 60,000 rpm!). Rotation of the laser spot has also been accomplished by more versatile means including a tip/tilt mirror [11], an acoustic-optical deflection system [17], or a pair of galvanometer-driven mirrors [18]. Among these schemes, a dual-galvanometer system has advantages in that it is relatively simple and inexpensive to implement, enables fast (several hundred fps) imaging synchronized with camera acquisition, and allows the excitation mode (azimuthal scan angle) to be changed ‘on the fly’ on a frame by frame basis. The system described by Lin & Hopppe [18] was designed specifically for FRET measurements, and features rapid changes in scan angle synchronized with alternating laser excitations to compensate for differences in TIRF penetration depth at different wavelengths. Our system is conceptually similar, but is optimized for fast, dynamic imaging of cellular Ca^2+^ signaling. Moreover, it can be readily constructed using commercially available, off-the-shelf components, and we make freely available custom software based on open source platforms for both controlling the scan mirrors, laser excitation and image acquisition and analysis software for de-interlacing and processing image data acquired with interleaved mode switching.

A particular feature is that the ability to rapidly alternate the scanning mode enables depth information to be obtained by altering the scan radius to achieve different penetration depths of the evanescent field; or to alternate between TIRF excitation and ‘skimming plane’ or wide-field conical epifluorescence excitation to achieve effectively simultaneous imaging in each mode. Here, we applied this capability to investigate the axial distribution of Ca^2+^ puff sites within neuroblastoma cells. We find that sites are distributed over a range of distances from the plasma membrane, with roughly one half of sites generating fluorescence signals that are larger in TIRF than in WF mode. This complicates analysis of puff signals recorded exclusively by TIRF. We had previously assumed a fixed ‘yardstick’ measure of the fluorescence corresponding to the opening of a single IP_3_R channel by which to estimate how many channels opened during any given puff [6], whereas our results now indicate that the single-channel signal–as determined by TIRF microscopy–will vary with depth. Thus, a more accurate quantal dissection of puffs should be obtained by employing interleaved TIRF/WF imaging to exclusively select superficial puff sites.

In summary, we describe a simple and inexpensive TIRF illumination system that provides much more even illumination than conventional stationary-spot TIRF systems, and moreover enables fast, near simultaneous imaging in both TIRF and wide-field modes. Although our system was designed primarily for dynamic Ca^2+^ imaging, for which a fast acquisition rate and corresponding scan speed are essential, it would be equally applicable for imaging many other near-membrane cellular processes such as exocytosis, lipid raft dynamics, and protein diffusion and trafficking.

## Supporting Information

S1 Fig Graphical user interface (GUI) for the spinning-spot TIRF galvanometer driver. The circular scan of the laser spot is achieved by two galvanometer mirrors driven by sine and cosine voltage waveforms generated by a National Instruments DAC card. The DAC signals are controlled using the GUI, written in Python. The frequency slider controls the frequency of the sine and cosine waves (i.e. the period of a complete circular scan). The radius slider controls the amplitude of both sine (x-deflection) and cosine (y deflection) waves. The ellipticity and phase sliders alter the relative amplitudes and phases of the two signals, respectively. The x-shift and y-shift sliders control the zero-offset of each signal (i.e. the center of the circular scan). The software further enables control of the on/off state and intensity of the lasers. Up to three different settings can be saved and recalled with the buttons in the lower panel. The GUI can alternate between up to three of these different settings during sequential cycles of the circular scan, enabling near simultaneous imaging in multiplexed modes.
(TIF)

S2 Fig Software for image processing and analysis. The screengrabs show the user interface for custom software used to process and analyze images. The software (FLIKA) was written in Python and can be run in the Linux, OSX, or Windows operating systems. (A) Image stacks saved in MetaMorph.stk file format or multi-plane TIFF format can be loaded and displayed. The user interface enables black level subtraction and pixel-by-pixel normalization relative to average baseline fluorescence, and de-interleaving. (B) The de-interleave window enables separation image stacks interleaving 2 or 3 imaging modalities into separate stacks. (C) Illustration of two de-interleaved image stacks, showing ratio (ΔF/F_0_) images with frames acquired alternately in TIRF and WF modes. Both image stacks can be scrolled in synchrony. Custom regions of interest (ROIs) can be drawn on one stack and mirrored in another, such that moving and resizing one produces the same changes in the other. (D) The average fluorescence intensity over time within mirrored regions of interest in each image stack can be overlaid and plotted in different colors to facilitate comparison. These traces can be exported into ASCII files.
(TIF)

S1 Movie Simultaneous imaging of fluorescent beads in suspension by TIRF and wide-field excitation. Aqueous suspension of fluorescent microspheres (0.5 μm diameter) imaged by alternating shadowless TIRF and widefield excitation. Images were acquired by alternating 5 ms exposures in each mode. The montage shows de-interlaced images (widefield, *left*; TIRF, *right*) after averaging 2 sequential frames. Three stationary microspheres had adhered to the coverglass, and others can be seen undergoing Brownian motion in the water. Image is 51 x 51 μm.
(MP4)

S2 Movie Multi-mode imaging of STIM/Orai relocalization following depletion of ER Ca^2+^ stores. Images in each panel of the video show EGFP-tagged Orai visualized by shadowless TIRF excitation at 488 nm (top left); mCherry-tagged STIM visualized by TIRF excitation at 532 nm (top right); mCherry-tagged STIM visualized by skimming plane (SP) excitation at 532 nm (bottom left); and a pseudocolored overlay (Orai in green, TIRF-STIM in red, SP-STIM in blue) of these three images. The imaging modes were alternated with 100 ms exposures in each mode. Thapsigargin was applied shortly after the beginning of the record to deplete ER Ca^2+^ and induce STIM and Orai relocalization. Each panel is 81 x 61 μm.
(MP4)

S1 Supporting Information Spinning-spot TIRF illuminator–Components List. (DOCX)
