# Supplementary figures and images for: Spinning-Spot Shadowless TIRF Microscopy
Source: PLoS One. 2015 Aug 26;10(8):e0136055. doi: 10.1371/journal.pone.0136055 (PMC4550233; doi:10.1371/journal.pone.0136055)

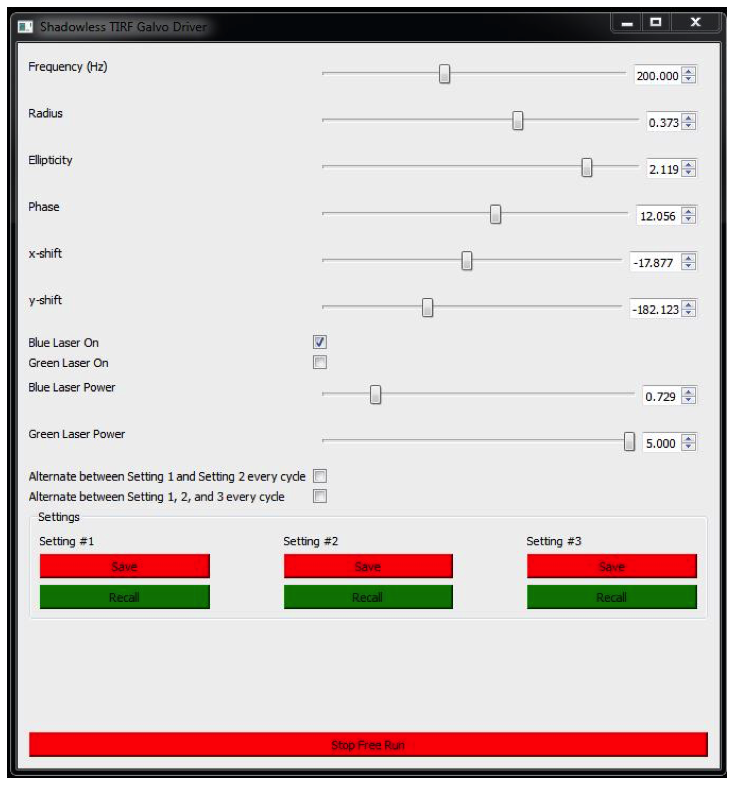

Supplement: S1 Fig — The circular scan of the laser spot is achieved by two galvanometer mirrors driven by sine and cosine voltage waveforms generated by a National Instruments DAC card. The DAC signals are controlled using the GUI, written in Python. The frequency slider controls the frequency of the sine and cosine waves (i.e. the period of a complete circular scan). The radius slider controls the amplitude of both sine (x-deflection) and cosine (y deflection) waves. The ellipticity and phase sliders alter the relative amplitudes and phases of the two signals, respectively. The x-shift and y-shift sliders control the zero-offset of each signal (i.e. the center of the circular scan). The software further enables control of the on/off state and intensity of the lasers. Up to three different settings can be saved and recalled with the buttons in the lower panel. The GUI can alternate between up to three of these different settings during sequential cycles of the circular scan, enabling near simultaneous imaging in multiplexed modes. (TIF) [file pone.0136055.s001.tif]

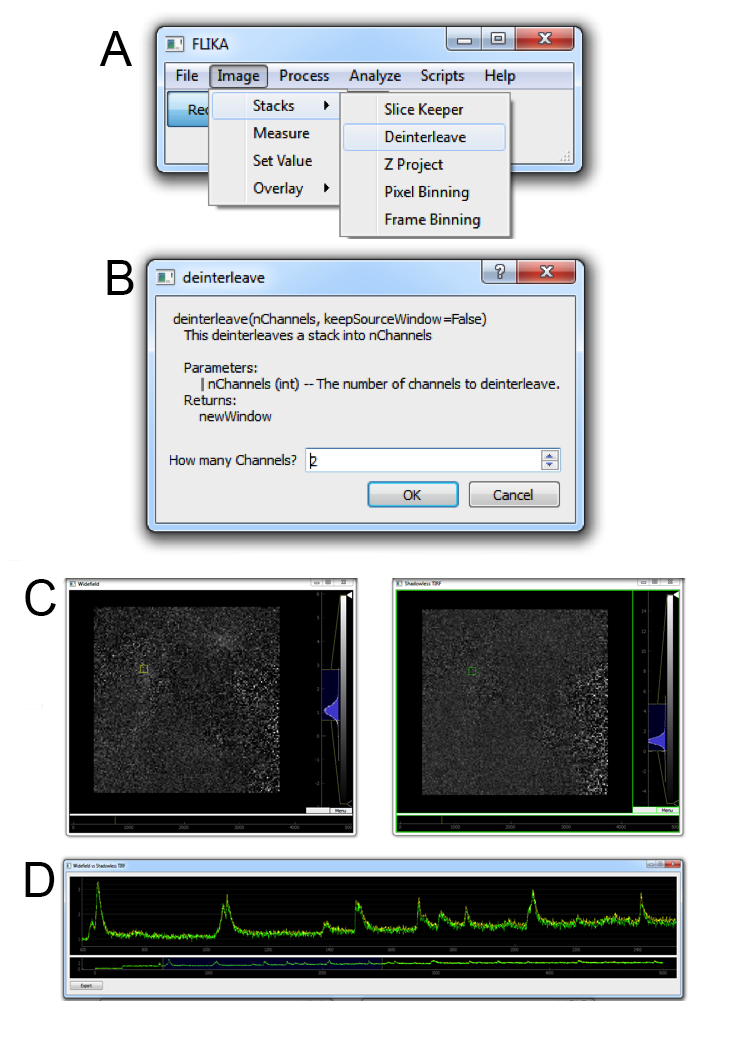

Supplement: S2 Fig — The screengrabs show the user interface for custom software used to process and analyze images. The software (FLIKA) was written in Python and can be run in the Linux, OSX, or Windows operating systems. (A) Image stacks saved in MetaMorph.stk file format or multi-plane TIFF format can be loaded and displayed. The user interface enables black level subtraction and pixel-by-pixel normalization relative to average baseline fluorescence, and de-interleaving. (B) The de-interleave window enables separation image stacks interleaving 2 or 3 imaging modalities into separate stacks. (C) Illustration of two de-interleaved image stacks, showing ratio (ΔF/F0) images with frames acquired alternately in TIRF and WF modes. Both image stacks can be scrolled in synchrony. Custom regions of interest (ROIs) can be drawn on one stack and mirrored in another, such that moving and resizing one produces the same changes in the other. (D) The average fluorescence intensity over time within mirrored regions of interest in each image stack can be overlaid and plotted in different colors to facilitate comparison. These traces can be exported into ASCII files. (TIF) [file pone.0136055.s002.tif]
