# Supplementary material for: Spinning-Spot Shadowless TIRF Microscopy
Source: PLoS One. 2015 Aug 26;10(8):e0136055. doi: 10.1371/journal.pone.0136055 (PMC4550233; doi:10.1371/journal.pone.0136055)
Supplement: S1 Supporting Information — (DOCX) [file pone.0136055.s005.docx]

**Spinning-spot TIRF illuminator – Components List**

**Laser and fiber launch**

RMS 10X Olympus Pan achromat objective

MBT613D Fiber launch with FC connector

P1-460B-FC-1 Single-mode fiber patch cable

KM100-E02 2x kinematic mount with visible laser mirror

UPH2 2x Universal ½ inch post holder

TR2 2x Optical post

RC08APC-P01 Reflective collimator

Laser; e.g. Coherent Obis 488 nm (<http://www.coherent.com/products/?1934/OBIS>)

OR the above fiber launch system could be replaced by using a fiber pigtailed laser, e.g. (<http://www.coherent.com/products/?2015/OBIS-Fiber-Pigtailed-FP>)

**Components required to add an additional laser**

KM100-E02 kinematic mount with visible laser mirror

UPH2 2x Universal ½ inch post holder

TR2 2x Optical post

KM100 Kinematic mount for laser beam combiner

Semrock laser MUX filter (<http://www.semrock.com/combining-or-separating-laser-beams.aspx>)

OR use Coherent OBIS Galaxy beam combiner with pigtailed fiber-coupled OBIS lasers (www.coherent.com/products/?2080/OBIS-Galaxy-and-OBIS-FP-Laser)

**Spinning spot fiber-coupled TIRF illuminator**

RC08APC-P01 Reflective collimator

ER4P4 Cage assembly rod; 4 inch (4 pack)

ER2-P4 Cage assembly rod; 2 inch (4 pack)

CP11 Cage plate; SM05 threaded

CP02T 2x Cage plate; SM-1 threaded

CP06 Cage plate for 1 inch optics

RSH4 2x 1inch post holder; 4 inch

PF175 2x mounting fork

RS4 2x 1 inch post; 4 inch

GCM002 Dual axis Galvo cage mount

GVSM002 2D Galvo system with accessories

SKU:OFPLWF10x 10x Olympus focusing eyepiece ([www.microscopeworld.com](http://www.microscopeworld.com))

50 mm f1.8 SLR camera lens

All components are from Thor Labs (<http://www.thorlabs.us/navigation.cfm>) unless otherwise noted.
